# Faster Increases in Human Life Expectancy Could Lead to Slower Population Aging

**DOI:** 10.1371/journal.pone.0121922

**Published:** 2015-04-15

**Authors:** Warren C. Sanderson, Sergei Scherbov

**Affiliations:** 1 Department of Economics, Stony Brook University, Stony Brook, New York, United States of America; 2 World Population Program, International Institute for Applied Systems Analysis, Laxenburg, Austria; 3 Wittgenstein Centre for Demography and Global Human Capital (IIASA, VID/ÖAW, WU), IIASA, Laxenburg, Austria; City University of New York, UNITED STATES

## Abstract

Counterintuitively, faster increases in human life expectancy could lead to slower population aging. The conventional view that faster increases in human life expectancy would lead to faster population aging is based on the assumption that people become old at a fixed chronological age. A preferable alternative is to base measures of aging on people’s time left to death, because this is more closely related to the characteristics that are associated with old age. Using this alternative interpretation, we show that faster increases in life expectancy would lead to slower population aging. Among other things, this finding affects the assessment of the speed at which countries will age.

## Introduction

Human population aging is a multidimensional phenomenon. If differs from aging at the level of individuals. Each additional year individuals are alive, they grow one year older, but defining how populations age is much more complex [1–4]. The study of human population aging can be divided into three components. The first is the change in the age structure of the population and the reasons for its change such as fertility and mortality. Age structure is usually measured using a few conventional measures such as the proportion of the population 65+ years old and the median age of the population. The second component is the change in the age-specific characteristics of people. For example, 65 year olds in the future are likely to have longer life expectancies, have higher average levels of education, and have better cognition than 65 year olds today. In some countries, 65 year olds today would be eligible for a full public pension, but 65 year olds in the future would not be. Older people in the future will have levels of many characteristics exhibited by younger people today. The third component of the study of human population aging is the interaction between changes in age structures and changes in the age-specific characteristics of people.

Population aging is widely discussed [5,6], but those discussions usually focus solely on age structure and omit consideration of the changing age-specific characteristics of people. An example of this is the United Nations’ *World Population Ageing 2013* [6], which provides much of the data currently used in studies of population aging. Those data, which are computed for all countries of the world at 5-year intervals from 1950 to 2100, are based solely on age structures. In that volume, the UN categorizes people as being old when they reach age 60. It does not matter that 60 year olds in 1950 are likely to be very different from 60 year olds in 2100.

Many studies of population aging focus on specific countries and make forecasts over many decades. Over these long spans of time of characteristics of older people can change considerably. 60 year olds, for example, could have become healthier [7], have longer life expectancies [6,8–10], have better cognition [11] and have become less dependent upon others for their daily care [7]. Sixty-five is sometimes used as an old age threshold, because in some countries it was the age at which some people could be eligible for a full state pension. However, in most OECD countries normal pension ages are now in the process of changing. For example, the normal pension age in the United States used to be 65. Now it is 66 and it is scheduled to rise to 67. Assessing the pension burden based on a fixed age of 65 would produce a misleading result.

In the US, Medicare, a form of national health insurance for older people, begins covering people at age 65. In most other OECD countries, 65 is not an especially important age in terms of health care costs paid by the government. Health care costs depend on age, but they are much higher in the last years of life [12]. As life expectancy increases, those last years of life happen later and later. Ignoring this could produce distorted figures. Analyses of the speed of population aging that disregard the changing characteristics of people are incomplete and potentially, for that reason, they can produce biased findings.

The first step in our analysis is the measurement of the speed of population aging using two measures that take into account changes in age structure, changes in the characteristics of people, and the interaction of those two. We call these the prospective proportion of the people who are old and the prospective median age. The second step is the comparison of the speed of aging using these more complete measures with the speed of aging using the conventional proportion of the population who are considered old and the conventional median age. The final step is the discussion and interpretation of our findings.

Ryder [13] challenged the conventional view in which people are classified as old based on a fixed chronological age. In the study of aging, he argued that it would be preferable to consider people as old not based on their chronological ages, but instead on their expected remaining lifetimes. He argued that those expected lifespans were a better reflection of older people’s health, dependence on others, and their general level of functioning than their chronological ages. Ryder’s insight has been independently rediscovered [1,14], applied [15–17] and elaborated [1–4,18,19]. This paper is based on those elaborations. In particular, here we use a measure of age, called prospective age, which is based on remaining life expectancy. That literature demonstrates that, in the study of population aging over a long time period and across countries with quite different mortality conditions, it is preferable to use a definition of age based on remaining life expectancy than one based on chronological age.

In this paper, we show a previously unobserved implication of the use of prospective age instead of chronological age. When prospective age is used, increases in measures of population aging are slower when the pace of life expectancy improvements is faster. This is the opposite of the conventional view that faster increases in life expectancy will cause the speed of aging to increase.

## Methods

The population projections made in this paper use the cohort component method [20] The required inputs are initial distributions of populations by age and sex in the base year and assumptions about future age-specific rates of fertility, mortality, and migration. In Scenario 3, we use the initial population distributions and the future age-specific rates that were used in making the projections in the European Demographic Data Sheet 2014 [21]. The initial population age structures used there come from Eurostat [22]. The forecasts of age-specific rates come from a study that utilized both historical data and expert opinion [23]. Details of the methodology can be found at European Demographic Data Sheet 2014: Learn more about the data, methods and assumptions used in the population projections [24]. Detailed notes can be found at the Vienna Institute of Demography website [25] by clicking on the link to “Sources and notes”.

Scenario 1 uses the same initial population distributions, age-specific fertility and migration assumptions as Scenario 3, but keeps age-specific survival rates constant. Scenario 2 was created on the assumption that changes in life expectancy at birth from 2013 onward were half of those in Scenario 3. This was done separately for men and women. Age-specific mortality rates in Scenario 2 and 3 were obtained from scenarios specified with respect to life expectancy at birth, using a Brass relationship model [26]. Table 1 lists the assumptions for all three scenarios.

**Table 1:** List of Assumptions for all Three Scenarios.

Scenario	Fertility	Mortality	Migration
1	Same as EDDS	No increase in life expectancy at birth	Same as EDDS
2	Same as EDDS	Increase in life expectancy at birth is half that assumed in the EDDS	Same as EDDS
3	Same as EDDS	Same as EDDS	Same as EDDS

Note: EDDS: European Demographic Data Sheet 2014 [21].

We compare the speed of population aging across those three scenarios using four measures. Two of them measure changes in the proportion of the population who are “old”. In the conventional approach, the threshold age at which people as assumed to become “old” is fixed, using at age 65. In contrast, we define the prospective old age threshold, which takes changes in longevity into account is variable. Here, as in our previous papers (citations here), we use an old age threshold the age at which remaining life expectancy first falls below 15 years. This is roughly the remaining life expectancy of people in many low mortality countries in the 1960s-70s [27]. For example, using the prospective old age threshold French women would be classified as being old beginning at age 58.4 in 1900, at age 64.8 in 1956, and at age 74.6 in 2012 [27].

Prospective median ages are the ages in 2013 where remaining life expectancy is the same as at the median age in the indicated year. More formally, Let *ma*(*t*) be the median age of a population in year t, *e(a*,*t)* be remaining life expectancy at age *a* in year *t* and let the remaining life expectancy at the median age be *e*(*ma*(*t*),*t*). The prospective median age is the age in the base year (2013, in this case) with the same remaining life expectancy as observed at the median age in year *t* (*e* ^-1^(*e*(*ma*(*t*),*t*),2013)).

The threshold ages for being categorized as old are defined as those ages where remaining life expectancy is 15 years or less. Let *a*_*old*_ *(t)* be the old age threshold in year *t*. The old age threshold, then is defined as *a*_*old*_(*t*) = *e* ^-1^(15,*t*).

## Results

We use three scenarios for population projections (see Table 1). The baseline scenario (Scenario 3) is the one used elsewhere to make population forecasts from 2013 to 2050 for all European countries [21]. Scenario 1 is identical to Scenario 3 except that life expectancies at birth are kept constant at their 2013 levels. In Scenario 2, gains in life expectancy at birth from 2013 onwards are half as large as in Scenario 3. Thus, the speed of life expectancy increase rises with the scenario number. The derived life expectancies at age 65 for all European countries for 2013, 2030, and 2050 are presented in S1 Table. The average pace of increase of life expectancy at 65 in all European countries is around 0.7 years per decade in the second scenario and around 1.4 years per decade in the third.

Table 2 shows the proportions of the German population, for the years 2013, 2030, and 2050, who are old using as the old age threshold: (1) age 65, and (2) the age when remaining life expectancy first falls below 15 years. Figures were initially computed separately for men and women and then combined. Table 2 shows that faster gains in life expectancy increase the measured speed of aging using the conventional measure, but decrease it when changes in longevity are taken into account. In 2050, in Scenario 1, with no life expectancy increase, the proportion of the German population 65+ years old would be 0.278. With the expected increase in life expectancy, but unaltered fertility and mortality rates, the proportion grows to 0.329. In Scenario 3, more elderly people survive and the proportion 65+ increases. When prospective age is used, the threshold age at which people are categorized as old changes over time. The prospective proportion old is 0.237 in Scenario 1. In Scenarios 2 and 3 it is lower, 0.218 and 0.199 respectively. The proportion of the population old falls with increases the speed of life expectancy gains.

**Table 2:** Conventional and Prospective Proportions Old, Germany 2013, 2030, and 2050: Three Scenarios of Life Expectancy Increase (both sexes).

	Proportion of Population 65+ Years Old (Conventional Proportion Old)			Proportion of Population in Age Groups with Remaining Life Expectancy of 15 Years or Less (Prospective Proportion Old)		
Year	Scenario 1	Scenario 2	Scenario 3	Scenario 1	Scenario 2	Scenario 3
**2013**	0.207	0.207	0.207	0.148	0.148	0.148
**2030**	0.267	0.273	0.279	0.177	0.166	0.156
**2050**	0.278	0.303	0.329	0.205	0.201	0.197

We provide data for only one country in the text because our focus is on the relationship between the speed of population aging and the speed of life expectancy change. If we had included data for several countries in Table 2, it would have shifted our focus to the differences between the countries. For completeness, we provide the same data in S2 Table. All the countries in S2 Table exhibit the same relationship between the measured speed of population aging and the speed of life expectancy change that we observe for Germany in Table 2.

The computation of the conventional and the prospective proportion old requires the specification of a criterion which separates old people from those who are not old. A common measure of population aging that does not require a separation criterion is the median age.

In Table 3, we show the conventional median age and prospective median age of women in Germany under the three scenarios for 2013, 2030, and 2050. The prospective median age is the age in the base year (in this case 2013) where remaining life expectancy is the same as at the median age in the indicated year. Thus, in addition to changes in the conventional median age, the prospective median age takes into account how life expectancy at the conventional median age is changing. As life expectancy increases it is possible that, simultaneously, the conventional median age grows older and remaining life expectancy at that median age grows longer [1]. Data are provided only for a single sex in order to simplify and clarify the presentation and the interpretation of the results. Figures for men and for both sexes combined tell exactly the same story.

**Table 3:** Conventional and Prospective Median Ages, Germany, 2013, 2030, and 2050 for 3 Scenarios of the Speed of Life Expectancy Increase (females).

	Conventional Median Age			Prospective Median Age		
**Year**	Scenario 1	Scenario 2	Scenario 3	Scenario 1	Scenario 2	Scenario 3
**2013**	46.5	46.5	46.5	46.5	46.5	46.5
**2030**	49.1	49.5	49.9	49.1	47.9	46.6
**2050**	49.3	50.9	52.6	49.3	47.4	45.6

Note: 2013 is used as the standard year.

The conventional median age and the prospective median age are identical in Scenario 1, where there is no increase in life expectancy. In 2013, the median age of the German population was 46.5 years. If age-specific survival rates remained constant at their 2013 levels, the median age is forecast to rise to 49.3 in 2050 because of the age structure of the German population in 2013 and assumptions about fertility and migration rates. If, in addition, age-specific survival rates were to increase as in Scenario 3, the median age would rise another 3.3 years to 52.6 in 2050. This example illustrates that when the conventional median age is used as an indicator of aging, faster increases in life expectancy appear to cause faster increases in aging.

When the prospective median age is used as an indicator of aging, Table 3 demonstrates the opposite result. If there would be no increase in life expectancy between 2013 and 2050, the prospective median age would rise 2.8 years from 46.5 to 49.3. If life expectancy increases, as in Scenario 3, the prospective median age actually decreases. The prospective median age in Scenario 3 in 2050 is 3.7 years lower than it would be under the assumption of no life expectancy increase. Here again faster increases in life expectancy lead to slower increases in measured population aging. This pattern, again, is the same for all European countries (S3 Table).

**Fig 1:**
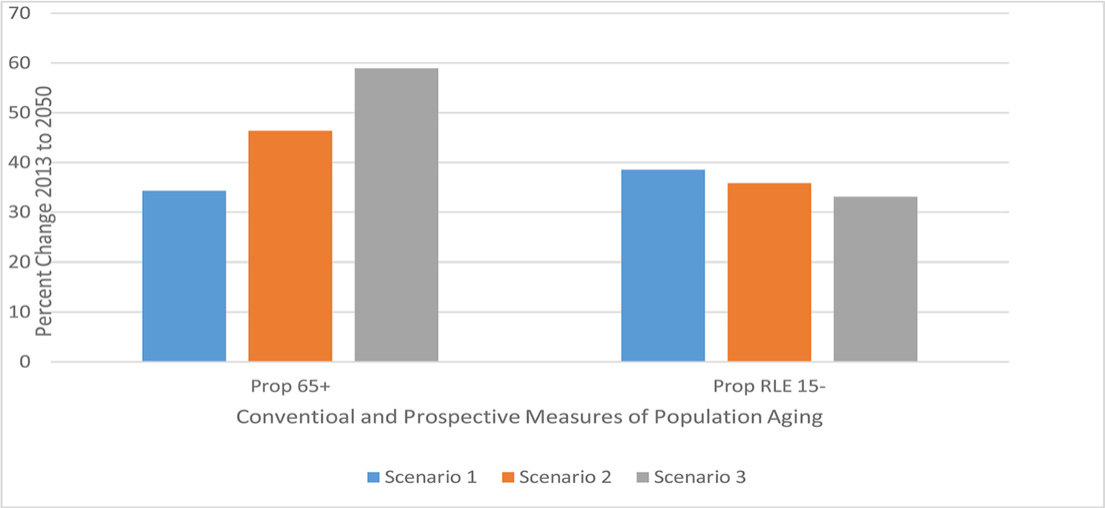
Percentage Increase in Proportions “Old” from 2013 to 2050, Using Measures Unadjusted and Adjusted for Longevity Change, Germany. Note: Prop 65+ is the proportion of the population 65+ years old. It is the conventional measure of the proportion of the population who are old. Prop. RLE 15- is the proportion of the population who are in age groups with remaining life expectancy of 15 years or less. It is the prospective measure of the proportion of the population who are “old”. The percentage increases in the proportions “old’ are one measure of the speed of aging. When the prospective measure of the speed of aging is used faster increases in life expectancy lead to slower increases in population aging.

**Fig 2:**
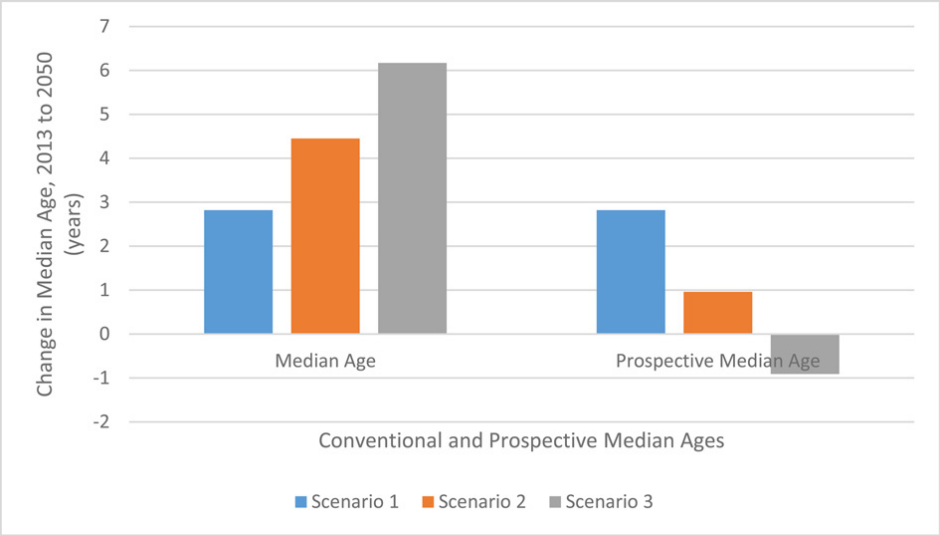
Changes in Median Age from 2013 to 2050 Using Measures Unadjusted and Adjusted for Longevity Increase, Germany. Note: See definition of prospective median age in the text. Changes in the median age are one measure of the speed of aging. When the prospective median age is used the speed of aging decreases when life expectancy increases is faster.

The main findings in Table 2 and Table 3 are summarized in Figs. 1 and 2. In Fig. 1, we show the forecasted relationship between the change in the proportion of the population who are categorized as being old and the speed of life expectancy change in Germany over the period 2013 to 2050. We show the relationship for the conventional measure of the proportion old where the old age threshold is fixed at age 65 and for the prospective measure where the old age threshold is defined as the age at which remaining life expectancy is 15 years. As the speed of life expectancy change increases (going upwards from Scenario 1 to Scenario 3), the change in the proportion of the population categorized as old always increases when the conventional measure is used, but always decreases when the prospective measure is used.

In Fig. 2, we show the forecasted relationship between the change in the median age of the population and the change in the speed of life expectancy change in Germany over the same period. The relationship is shown for the conventional median age and for the prospective median age. As the speed of life expectancy change increases (going upwards from Scenario 1 to Scenario 3), changes in the conventional median age increase, but changes in the prospective age decrease. In both Figures, it can be seen that faster increases in the speed of life expectancy result in slower changes in prospective measures of population aging. This is just the opposite of the result when conventional measures are used.

## Discussion

We present data here on the two of most commonly used measures of population aging, the proportion of the population old and the median age of the population for 39 European countries for 2013, 2030, and 2050 in S2 Table and S3 Table. These countries, which include Germany, Iceland, Moldova, and Russia, exhibit a wide variety of demographic conditions and in all these cases the prospective measures indicate that faster increases in life expectancy lead to slower population aging.

The main difference between the prospective and conventional measures of the proportion of the population who are old is not that they use different old age thresholds. It is that, in the case of the prospective measure, the old age threshold changes over time as life expectancy changes.

Both the conventional old age threshold, which is fixed at age 65, and the variable one used here have an element of arbitrariness. Conventional measures of population aging could categorize people as becoming old beginning at age 60, 65, or 70. Prospective measures could categorize people as becoming old, when they are in age groups with 10, 15, or 20 years of remaining life expectancy. The conclusions with respect to the relationship between the speed of life expectancy increase and the measured speed of population aging presented above are robust to any plausible changes in those values. For example, if we had computed tables showing the speed of aging using a fixed old age threshold of 60 and a prospective old age threshold of 10 remaining years of life, the major trends would have been the same.

The mechanics of the computation of prospective median age and the proportion old are quite different. The life table for each country in 2013 is used as a standard in the calculation of prospective median ages. It does not matter which year is chosen for the standard or even if one of the standards was used for all the countries. Faster increases in life expectancy would still lead to slower changes in measures of population aging.

The prospective median age is an indicator of the median remaining lifespan of the population. When we compare populations in Scenario 1, with no increase in longevity, with those in Scenario 3, we see that the chronological median age of the populations in Scenario 3 are higher than those in Scenario 1, but that the median remaining life expectancies are also higher. Increases in life expectancy gains make populations relatively younger in the sense of having a longer median remaining lifespan.

The connections between life expectancy and aging presented here are important for understanding the future speed of aging. There are numerous data sources that provide information on the extent and speed of aging in various countries [28,29]. Virtually all of these cite the UN measures that assume old age begins at 60 or 65, but those measures are incomplete. There are two aspects of aging that need to be incorporated into studies of population aging, changes in the age structures of populations and changes in the characteristics of people.

One way in which population aging can occur is that fertility falls and everything else about the population, including life expectancy, remains the same. This is a situation in which the age structure of the population changes, but the age-specific characteristics of people remain the same. In this case, the conventional and prospective proportions of the population who are old and the conventional and prospective median ages of the population would rise, not because there are more elderly people, but because there are fewer young people. Faster decreases in fertility, everything else being equal, would result in faster increases in the measures of aging, but conventional and prospective median ages would remain identical. When population aging happens only because of reductions in fertility, but not because of changes in life expectancy, the conventional measures, based solely on chronological age work well.

Population aging in most European countries in the last half century has not been the result solely of decreases in fertility. Life expectancy has also risen at each older age. The prospective approach takes this into account.

## Supporting Information

S1 Table Remaining Life Expectancies at Age 65 (females). Scenarios are based on the assumptions concerning life expectancies at birth discussed in the text.
(PDF)

S2 Table Conventional and Prospective Proportions Old (both sexes). Scenarios are based on the assumptions concerning life expectancies at birth discussed in the text.
(PDF)

S3 Table Median Age and Prospective Median Age, 3 scenarios (females). Scenarios are based on the assumptions concerning life expectancies at birth discussed in the text.
(PDF)
