# Supplementary material for: Faster Increases in Human Life Expectancy Could Lead to Slower Population Aging
Source: PLoS One. 2015 Apr 15;10(4):e0121922. doi: 10.1371/journal.pone.0121922 (PMC4398478; doi:10.1371/journal.pone.0121922)
Supplement: S1 Table — Scenarios are based on the assumptions concerning life expectancies at birth discussed in the text. (PDF) [file pone.0121922.s001.pdf]

Supporting Information

S1 Table. Remaining Life Expectancies at Age 65 (females).

	Scenario 1	Scenario 2	Scenario 3
**Albania**			
2013	19.43	19.43	19.43
2030	19.43	20.45	21.56
2050	19.43	21.74	24.44
**Austria**			
2013	21.37	21.37	21.37
2030	21.37	22.75	24.24
2050	21.37	24.47	28.01
**Belarus**			
2013	17.45	17.45	17.45
2030	17.45	18.23	19.07
2050	17.45	19.45	21.79
**Belgium**			
2013	21.31	21.31	21.31
2030	21.31	22.66	24.13
2050	21.31	24.12	27.35
**Bulgaria**			
2013	17.18	17.18	17.18
2030	17.18	17.95	18.78
2050	17.18	19.19	21.55
**Croatia**			
2013	18.68	18.68	18.68
2030	18.68	19.83	21.07
2050	18.68	21.25	24.21
**Cyprus**			
2013	20.41	20.41	20.41
2030	20.41	21.41	22.48
2050	20.41	22.97	25.86
**Czech Rep.**			
2013	19.32	19.32	19.32
2030	19.32	20.67	22.12
2050	19.32	22.32	25.77
**Denmark**			
2013	20.17	20.17	20.17
2030	20.17	21.20	22.30
2050	20.17	22.63	25.38

	Scenario 1	Scenario 2	Scenario 3
**Estonia**			
2013	20.00	20.00	20.00
2030	20.00	20.41	20.84
2050	20.00	21.67	23.53
**Finland**			
2013	21.61	21.61	21.61
2030	21.61	22.85	24.18
2050	21.61	24.33	27.43
**France**			
2013	23.70	23.70	23.70
2030	23.70	24.82	26.01
2050	23.70	26.30	29.22
**Georgia**			
2013	17.63	17.63	17.63
2030	17.63	18.41	19.24
2050	17.63	19.70	22.08
**Germany**			
2013	21.14	21.14	21.14
2030	21.14	22.33	23.60
2050	21.14	23.81	26.84
**Greece**			
2013	20.84	20.84	20.84
2030	20.84	22.20	23.66
2050	20.84	23.73	27.06
**Hungary**			
2013	18.09	18.09	18.09
2030	18.09	19.03	20.05
2050	18.09	20.27	22.81
**Iceland**			
2013	21.40	21.40	21.40
2030	21.40	22.73	24.13
2050	21.40	24.33	27.56
**Ireland**			
2013	20.99	20.99	20.99
2030	20.99	22.05	23.18
2050	20.99	23.49	26.30
**Italy**			
2013	22.61	22.61	22.61
2030	22.61	23.77	24.99
2050	22.61	25.33	28.35

	Scenario 1	Scenario 2	Scenario 3
**Latvia**			
2013	18.43	18.43	18.43
2030	18.43	19.33	20.30
2050	18.43	20.58	23.08
**Lithuania**			
2013	19.07	19.07	19.07
2030	19.07	19.89	20.77
2050	19.07	21.14	23.54
**Luxembourg**			
2013	21.24	21.24	21.24
2030	21.24	22.30	23.41
2050	21.24	23.79	26.64
**Macedonia**			
2013	15.93	15.93	15.93
2030	15.93	17.05	18.29
2050	15.93	18.52	21.68
**Malta**			
2013	20.64	20.64	20.64
2030	20.64	21.64	22.70
2050	20.64	23.08	25.87
**Moldova**			
2013	15.57	15.57	15.57
2030	15.57	16.18	16.83
2050	15.57	17.30	19.32
**Montenegro**			
2013	17.25	17.25	17.25
2030	17.25	18.64	20.19
2050	17.25	20.05	23.42
**Netherland**			
2013	21.05	21.05	21.05
2030	21.05	22.13	23.27
2050	21.05	23.58	26.43
**Norway**			
2013	21.24	21.24	21.24
2030	21.24	22.44	23.71
2050	21.24	23.96	27.00
**Poland**			
2013	19.73	19.73	19.73
2030	19.73	20.66	21.64
2050	19.73	21.99	24.55

	Scenario 1	Scenario 2	Scenario 3
**Portugal**			
2013	21.23	21.23	21.23
2030	21.23	22.29	23.42
2050	21.23	23.75	26.62
**Romania**			
2013	17.71	17.71	17.71
2030	17.71	18.80	20.00
2050	17.71	20.11	22.96
**Russian Federation**			
2013	16.97	16.97	16.97
2030	16.97	17.59	18.26
2050	16.97	18.54	20.39
**Serbia**			
2013	16.49	16.49	16.49
2030	16.49	17.39	18.36
2050	16.49	18.67	21.24
**Slovakia**			
2013	18.44	18.44	18.44
2030	18.44	19.49	20.62
2050	18.44	20.85	23.65
**Slovenia**			
2013	20.86	20.86	20.86
2030	20.86	21.83	22.85
2050	20.86	23.28	25.98
**Spain**			
2013	22.64	22.64	22.64
2030	22.64	23.62	24.66
2050	22.64	25.15	27.97
**Sweden**			
2013	21.22	21.22	21.22
2030	21.22	22.33	23.50
2050	21.22	23.83	26.72
**Switzerland**			
2013	22.42	22.42	22.42
2030	22.42	23.82	25.31
2050	22.42	25.37	28.71
**UK**			
2013	21.22	21.22	21.22
2030	21.22	22.34	23.52
2050	21.22	23.76	26.62

	Scenario 1	Scenario 2	Scenario 3
**Ukraine**			
2013	16.65	16.65	16.65
2030	16.65	16.95	17.26
2050	16.65	17.96	19.44

Note: Scenarios are based on the assumptions concerning life expectancies at birth discussed in the text.
