# Supplementary material for: Faster Increases in Human Life Expectancy Could Lead to Slower Population Aging
Source: PLoS One. 2015 Apr 15;10(4):e0121922. doi: 10.1371/journal.pone.0121922 (PMC4398478; doi:10.1371/journal.pone.0121922)
Supplement: S2 Table — Scenarios are based on the assumptions concerning life expectancies at birth discussed in the text. (PDF) [file pone.0121922.s002.pdf]

Supporting Information

S2 Table. Proportions Old (both sexes combined).

	Proportion 65+			Proportion With Remaining Life Expectancy 15 Years or Less		
	Scenario 1	Scenario 2	Scenario 3	Scenario 1	Scenario 2	Scenario 3
**Albania**						
2013	0.118	0.118	0.118	0.099	0.099	0.099
2030	0.189	0.193	0.198	0.154	0.145	0.135
2050	0.222	0.242	0.263	0.190	0.179	0.170
**Austria**						
2013	0.181	0.181	0.181	0.119	0.119	0.119
2030	0.236	0.243	0.250	0.152	0.141	0.131
2050	0.263	0.290	0.317	0.189	0.183	0.174
**Belarus**						
2013	0.138	0.138	0.138	0.148	0.148	0.148
2030	0.197	0.201	0.205	0.198	0.185	0.174
2050	0.224	0.244	0.267	0.231	0.209	0.190
**Belgium**						
2013	0.176	0.176	0.176	0.121	0.121	0.121
2030	0.216	0.222	0.228	0.146	0.137	0.128
2050	0.220	0.241	0.263	0.158	0.152	0.145
**Bulgaria**						
2013	0.192	0.192	0.192	0.182	0.182	0.182
2030	0.227	0.231	0.235	0.218	0.209	0.201
2050	0.260	0.281	0.304	0.250	0.235	0.220
**Croatia**						
2013	0.181	0.181	0.181	0.158	0.158	0.158
2030	0.228	0.234	0.241	0.197	0.187	0.176
2050	0.245	0.269	0.295	0.212	0.198	0.185
**Cyprus**						
2013	0.132	0.132	0.132	0.087	0.087	0.087
2030	0.169	0.172	0.175	0.115	0.109	0.104
2050	0.197	0.211	0.226	0.133	0.123	0.113
**Czech Rep.**						
2013	0.168	0.168	0.168	0.126	0.126	0.126
2030	0.210	0.217	0.223	0.173	0.164	0.155
2050	0.239	0.264	0.290	0.197	0.180	0.159

	Proportion 65+			Proportion With Remaining Life Expectancy 15 Years or Less		
	Scenario 1	Scenario 2	Scenario 3	Scenario 1	Scenario 2	Scenario 3
**Denmark**						
2013	0.178	0.178	0.178	0.117	0.117	0.117
2030	0.212	0.215	0.219	0.154	0.147	0.140
2050	0.199	0.215	0.233	0.153	0.148	0.140
**Estonia**						
2013	0.180	0.180	0.180	0.147	0.147	0.147
2030	0.222	0.224	0.225	0.181	0.176	0.170
2050	0.244	0.258	0.273	0.202	0.189	0.177
**Finland**						
2013	0.188	0.188	0.188	0.117	0.117	0.117
2030	0.238	0.244	0.250	0.167	0.159	0.151
2050	0.217	0.237	0.258	0.152	0.146	0.141
**France**						
2013	0.175	0.175	0.175	0.103	0.103	0.103
2030	0.228	0.233	0.237	0.137	0.129	0.122
2050	0.233	0.251	0.271	0.153	0.147	0.139
**Georgia**						
2013	0.138	0.138	0.138	0.132	0.132	0.132
2030	0.195	0.199	0.204	0.176	0.166	0.155
2050	0.257	0.279	0.302	0.237	0.218	0.199
**Germany**						
2013	0.207	0.207	0.207	0.148	0.148	0.148
2030	0.267	0.273	0.279	0.177	0.166	0.156
2050	0.278	0.303	0.329	0.205	0.201	0.197
**Greece**						
2013	0.201	0.201	0.201	0.144	0.144	0.144
2030	0.234	0.240	0.247	0.164	0.155	0.146
2050	0.280	0.304	0.330	0.205	0.192	0.178
**Hungary**						
2013	0.172	0.172	0.172	0.153	0.153	0.153
2030	0.208	0.213	0.219	0.191	0.183	0.176
2050	0.239	0.261	0.283	0.219	0.206	0.192
**Iceland**						
2013	0.129	0.129	0.129	0.076	0.076	0.076
2030	0.181	0.185	0.189	0.111	0.105	0.098
2050	0.191	0.206	0.223	0.124	0.119	0.113
**Ireland**						
2013	0.122	0.122	0.122	0.079	0.079	0.079
2030	0.169	0.172	0.176	0.113	0.107	0.101
2050	0.211	0.226	0.242	0.147	0.136	0.125

	Proportion 65+			Proportion With Remaining Life Expectancy 15 Years or Less		
	Scenario 1	Scenario 2	Scenario 3	Scenario 1	Scenario 2	Scenario 3
**Italy**						
2013	0.212	0.212	0.212	0.137	0.137	0.137
2030	0.261	0.266	0.271	0.166	0.158	0.150
2050	0.302	0.325	0.347	0.218	0.206	0.192
**Latvia**						
2013	0.188	0.188	0.188	0.173	0.173	0.173
2030	0.226	0.231	0.237	0.203	0.192	0.181
2050	0.252	0.275	0.300	0.232	0.215	0.201
**Lithuania**						
2013	0.182	0.182	0.182	0.160	0.160	0.160
2030	0.228	0.232	0.236	0.193	0.182	0.170
2050	0.244	0.265	0.288	0.217	0.205	0.195
**Luxembourg**						
2013	0.140	0.140	0.140	0.094	0.094	0.094
2030	0.172	0.175	0.179	0.110	0.103	0.097
2050	0.186	0.202	0.218	0.130	0.124	0.118
**Macedonia**						
2013	0.120	0.120	0.120	0.122	0.122	0.122
2030	0.170	0.176	0.182	0.172	0.162	0.152
2050	0.210	0.235	0.261	0.212	0.194	0.178
**Malta**						
2013	0.172	0.172	0.172	0.109	0.109	0.109
2030	0.238	0.242	0.246	0.177	0.168	0.160
2050	0.252	0.270	0.289	0.182	0.168	0.157
**Moldova**						
2013	0.099	0.099	0.099	0.115	0.115	0.115
2030	0.180	0.183	0.186	0.196	0.187	0.178
2050	0.256	0.275	0.296	0.290	0.257	0.227
**Montenegro**						
2013	0.132	0.132	0.132	0.119	0.119	0.119
2030	0.182	0.188	0.195	0.164	0.153	0.143
2050	0.200	0.223	0.249	0.180	0.167	0.154
**Netherland**						
2013	0.168	0.168	0.168	0.107	0.107	0.107
2030	0.234	0.239	0.244	0.160	0.152	0.143
2050	0.238	0.258	0.279	0.178	0.173	0.167
**Norway**						
2013	0.157	0.157	0.157	0.097	0.097	0.097
2030	0.190	0.194	0.199	0.128	0.121	0.115
2050	0.198	0.214	0.232	0.140	0.133	0.125

	Proportion 65+			Proportion With Remaining Life Expectancy 15 Years or Less		
	Scenario 1	Scenario 2	Scenario 3	Scenario 1	Scenario 2	Scenario 3
**Poland**						
2013	0.142	0.142	0.142	0.112	0.112	0.112
2030	0.215	0.220	0.224	0.174	0.165	0.156
2050	0.259	0.279	0.300	0.203	0.184	0.168
**Portugal**						
2013	0.194	0.194	0.194	0.136	0.136	0.136
2030	0.238	0.243	0.248	0.167	0.159	0.150
2050	0.261	0.281	0.303	0.195	0.184	0.172
**Romania**						
2013	0.164	0.164	0.164	0.149	0.149	0.149
2030	0.210	0.216	0.222	0.193	0.185	0.176
2050	0.271	0.297	0.324	0.251	0.236	0.218
**Russian Federation**						
2013	0.129	0.129	0.129	0.138	0.138	0.138
2030	0.184	0.187	0.190	0.187	0.178	0.169
2050	0.199	0.213	0.230	0.206	0.189	0.172
**Serbia**						
2013	0.176	0.176	0.176	0.173	0.173	0.173
2030	0.217	0.223	0.229	0.212	0.204	0.195
2050	0.218	0.240	0.263	0.213	0.199	0.185
**Slovakia**						
2013	0.131	0.131	0.131	0.113	0.113	0.113
2030	0.195	0.200	0.206	0.172	0.162	0.153
2050	0.240	0.262	0.286	0.212	0.194	0.176
**Slovenia**						
2013	0.171	0.171	0.171	0.123	0.123	0.123
2030	0.239	0.243	0.246	0.174	0.165	0.157
2050	0.271	0.290	0.310	0.203	0.190	0.178
**Spain**						
2013	0.177	0.177	0.177	0.114	0.114	0.114
2030	0.230	0.234	0.238	0.142	0.135	0.127
2050	0.300	0.320	0.340	0.207	0.192	0.175
**Sweden**						
2013	0.191	0.191	0.191	0.119	0.119	0.119
2030	0.209	0.214	0.218	0.145	0.139	0.133
2050	0.200	0.217	0.234	0.142	0.135	0.128
**Switzerland**						
2013	0.174	0.174	0.174	0.102	0.102	0.102
2030	0.233	0.239	0.245	0.140	0.131	0.122
2050	0.260	0.282	0.305	0.175	0.168	0.159

	Proportion 65+			Proportion With Remaining Life Expectancy 15 Years or Less		
	**Scenario 1**	**Scenario 2**	**Scenario 3**	**Scenario 1**	**Scenario 2**	**Scenario 3**
**UK**						
2013	0.172	0.172	0.172	0.109	0.109	0.109
2030	0.206	0.211	0.215	0.136	0.129	0.122
2050	0.215	0.232	0.249	0.150	0.143	0.137
**Ukraine**						
2013	0.152	0.152	0.152	0.162	0.162	0.162
2030	0.190	0.192	0.193	0.196	0.192	0.187
2050	0.214	0.226	0.238	0.225	0.209	0.192

Note: Scenarios are based on the assumptions concerning life expectancies at birth discussed in the text.
