# Supplementary material for: Faster Increases in Human Life Expectancy Could Lead to Slower Population Aging
Source: PLoS One. 2015 Apr 15;10(4):e0121922. doi: 10.1371/journal.pone.0121922 (PMC4398478; doi:10.1371/journal.pone.0121922)
Supplement: S3 Table — Scenarios are based on the assumptions concerning life expectancies at birth discussed in the text. (PDF) [file pone.0121922.s003.pdf]

Supporting Information

S3 Table. Median Age and Prospective Median Age, 3 scenarios (females).

	Median Age			Prospective Median Age		
	Scenario 1	Scenario 2	Scenario 3	Scenario 1	Scenario 2	Scenario 3
**Albania**						
2013	35.63	35.63	35.63	35.63	35.63	35.63
2030	42.09	42.33	42.57	42.09	40.99	39.84
2050	50.68	51.59	52.55	50.68	48.75	46.59
**Austria**						
2013	43.80	43.80	43.80	43.80	43.80	43.80
2030	47.07	47.45	47.83	47.07	45.62	44.12
2050	48.51	50.26	52.07	48.51	46.29	44.00
**Belarus**						
2013	42.32	42.32	42.32	42.32	42.32	42.32
2030	46.53	46.72	46.91	46.53	45.45	44.36
2050	48.44	50.05	51.77	48.44	47.01	45.57
**Belgium**						
2013	42.22	42.22	42.22	42.22	42.22	42.22
2030	43.40	43.78	44.17	43.40	41.88	40.35
2050	43.21	44.53	45.97	43.21	40.72	38.25
**Bulgaria**						
2013	44.96	44.96	44.96	44.96	44.96	44.96
2030	48.91	49.18	49.45	48.91	47.98	47.03
2050	49.69	51.26	53.16	49.69	48.28	47.09
**Croatia**						
2013	44.52	44.52	44.52	44.52	44.52	44.52
2030	47.18	47.57	47.96	47.18	45.95	44.71
2050	48.40	50.05	51.70	48.40	46.58	44.68
**Cyprus**						
2013	37.10	37.10	37.10	37.10	37.10	37.10
2030	41.85	42.00	42.14	41.85	40.71	39.54
2050	44.20	45.22	46.27	44.20	42.03	39.77
**Czech Rep.**						
2013	41.93	41.93	41.93	41.93	41.93	41.93
2030	46.88	47.29	47.70	46.88	45.42	43.92
2050	45.57	47.36	49.28	45.57	43.32	41.12
**Denmark**						
2013	41.90	41.90	41.90	41.90	41.90	41.90
2030	42.09	42.39	42.68	42.09	40.94	39.77
2050	41.48	42.55	43.66	41.48	39.21	36.91

	Median Age			Prospective Median Age		
	Scenario 1	Scenario 2	Scenario 3	Scenario 1	Scenario 2	Scenario 3
**Estonia**						
2013	44.29	44.29	44.29	44.29	44.29	44.29
2030	47.26	47.33	47.40	47.26	46.73	46.19
2050	47.32	48.33	49.41	47.32	46.00	44.63
**Finland**						
2013	44.04	44.04	44.04	44.04	44.04	44.04
2030	44.29	44.68	45.06	44.29	43.01	41.69
2050	42.51	43.81	45.23	42.51	40.22	37.96
**France**						
2013	41.83	41.83	41.83	41.83	41.83	41.83
2030	43.85	44.13	44.42	43.85	42.59	41.33
2050	43.63	44.78	46.01	43.63	41.33	39.05
**Georgia**						
2013	39.69	39.69	39.69	39.69	39.69	39.69
2030	45.99	46.17	46.35	45.99	45.03	44.04
2050	51.78	53.43	54.99	51.78	50.61	49.21
**Germany**						
2013	46.46	46.46	46.46	46.46	46.46	46.46
2030	49.07	49.46	49.85	49.07	47.85	46.61
2050	49.28	50.91	52.63	49.28	47.42	45.55
**Greece**						
2013	43.80	43.80	43.80	43.80	43.80	43.80
2030	48.58	48.96	49.33	48.58	47.19	45.75
2050	48.34	50.07	51.93	48.34	46.41	44.49
**Hungary**						
2013	43.47	43.47	43.47	43.47	43.47	43.47
2030	47.76	48.12	48.46	47.76	46.60	45.41
2050	48.46	49.93	51.50	48.46	46.56	44.69
**Iceland**						
2013	36.19	36.19	36.19	36.19	36.19	36.19
2030	38.53	38.77	39.00	38.53	37.09	35.58
2050	39.87	40.81	41.81	39.87	37.20	34.43
**Ireland**						
2013	35.90	35.90	35.90	35.90	35.90	35.90
2030	40.57	40.83	41.07	40.57	39.37	38.14
2050	40.75	41.59	42.46	40.75	38.27	35.74
**Italy**						
2013	45.71	45.71	45.71	45.71	45.71	45.71
2030	51.08	51.38	51.69	51.08	49.94	48.77
2050	50.54	52.16	53.86	50.54	48.84	47.12

	Median Age			Prospective Median Age		
	Scenario 1	Scenario 2	Scenario 3	Scenario 1	Scenario 2	Scenario 3
**Latvia**						
2013	45.68	45.68	45.68	45.68	45.68	45.68
2030	48.54	48.89	49.24	48.54	47.48	46.40
2050	50.88	52.87	54.85	50.88	49.75	48.47
**Lithuania**						
2013	45.15	45.15	45.15	45.15	45.15	45.15
2030	48.11	48.41	48.72	48.11	47.09	46.06
2050	50.64	52.18	53.73	50.64	49.07	47.40
**Luxembourg**						
2013	39.68	39.68	39.68	39.68	39.68	39.68
2030	40.30	40.49	40.67	40.30	39.04	37.79
2050	40.47	41.31	42.17	40.47	37.97	35.40
**Macedonia**						
2013	37.67	37.67	37.67	37.67	37.67	37.67
2030	42.89	43.20	43.52	42.89	41.51	40.07
2050	46.25	47.91	49.58	46.25	44.15	41.91
**Malta**						
2013	41.96	41.96	41.96	41.96	41.96	41.96
2030	45.79	46.05	46.30	45.79	44.74	43.63
2050	48.17	49.46	50.79	48.17	46.34	44.41
**Moldova**						
2013	36.78	36.78	36.78	36.78	36.78	36.78
2030	45.71	45.83	45.95	45.71	44.80	43.84
2050	55.62	56.66	57.65	55.62	53.99	52.17
**Montenegro**						
2013	38.50	38.50	38.50	38.50	38.50	38.50
2030	42.06	42.49	42.92	42.06	40.40	38.66
2050	43.95	45.56	47.18	43.95	41.48	38.91
**Netherland**						
2013	42.39	42.39	42.39	42.39	42.39	42.39
2030	44.90	45.22	45.55	44.90	43.72	42.53
2050	44.98	46.28	47.62	44.98	42.87	40.72
**Norway**						
2013	39.68	39.68	39.68	39.68	39.68	39.68
2030	40.52	40.78	41.04	40.52	39.19	37.83
2050	40.69	41.72	42.79	40.69	38.19	35.63
**Poland**						
2013	40.53	40.53	40.53	40.53	40.53	40.53
2030	46.63	46.83	47.03	46.63	45.47	44.29
2050	49.84	51.34	52.86	49.84	48.15	46.42

	Median Age			Prospective Median Age		
	Scenario 1	Scenario 2	Scenario 3	Scenario 1	Scenario 2	Scenario 3
**Portugal**						
2013	44.14	44.14	44.14	44.14	44.14	44.14
2030	48.91	49.18	49.46	48.91	47.78	46.62
2050	47.63	48.93	50.25	47.63	45.68	43.62
**Romania**						
2013	42.70	42.70	42.70	42.70	42.70	42.70
2030	49.00	49.34	49.70	49.00	47.70	46.36
2050	51.18	52.96	54.92	51.18	49.52	47.89
**Russian Federation**						
2013	41.24	41.24	41.24	41.24	41.24	41.24
2030	44.93	45.08	45.24	44.93	43.97	42.99
2050	44.80	45.88	47.03	44.80	43.18	41.52
**Serbia**						
2013	44.51	44.51	44.51	44.51	44.51	44.51
2030	46.48	46.80	47.13	46.48	45.40	44.30
2050	46.42	47.75	49.18	46.42	44.50	42.56
**Slovakia**						
2013	39.74	39.74	39.74	39.74	39.74	39.74
2030	45.85	46.13	46.41	45.85	44.59	43.30
2050	48.01	49.68	51.35	48.01	46.29	44.48
**Slovenia**						
2013	44.00	44.00	44.00	44.00	44.00	44.00
2030	48.25	48.49	48.73	48.25	47.17	46.08
2050	47.54	49.10	50.75	47.54	45.92	44.30
**Spain**						
2013	42.43	42.43	42.43	42.43	42.43	42.43
2030	49.23	49.41	49.59	49.23	48.13	47.03
2050	49.24	50.59	52.05	49.24	47.45	45.66
**Sweden**						
2013	41.91	41.91	41.91	41.91	41.91	41.91
2030	41.53	41.79	42.06	41.53	40.33	39.10
2050	40.47	41.46	42.49	40.47	38.07	35.66
**Switzerland**						
2013	42.93	42.93	42.93	42.93	42.93	42.93
2030	46.27	46.62	46.98	46.27	44.82	43.34
2050	46.53	48.08	49.68	46.53	44.38	42.15
**UK**						
2013	40.85	40.85	40.85	40.85	40.85	40.85
2030	42.41	42.67	42.93	42.41	41.12	39.83
2050	41.73	42.84	44.01	41.73	39.42	37.10

	Median Age			Prospective Median Age		
	Scenario 1	Scenario 2	Scenario 3	Scenario 1	Scenario 2	Scenario 3
**Ukraine**						
2013	42.89	42.89	42.89	42.89	42.89	42.89
2030	45.99	46.06	46.13	45.99	45.56	45.11
2050	46.13	47.08	48.12	46.13	44.94	43.76

Note: Scenarios are based on the assumptions concerning life expectancies at birth discussed in the text.
